# Comparative efficacy of surgical approaches to disease modification in Parkinson disease

**DOI:** 10.1038/s41531-022-00296-w

**Published:** 2022-03-25

**Authors:** Shervin Rahimpour, Su-Chun Zhang, Jerrold L. Vitek, Kyle T. Mitchell, Dennis A. Turner

**Affiliations:** 1 Department of Neurosurgery, Clinical Neuroscience Center, University of Utah, Salt Lake City, UT 84132 USA; 2 Waisman Center and Departments of Neuroscience and Neurology, University of Wisconsin-Madison, Madison, WI 53705 USA; 3 Program in Neuroscience & Behavioral Disorders, Duke-NUS Medical School, 169857 Singapore, Singapore; 4 Department of Neurology, University of Minnesota, Minneapolis, MN 55455 USA; 5 Department of Neurology, Duke University, Durham, NC 27710 USA; 6 Department of Neurosurgery, Duke University, Durham, NC 27710 USA; 7 Department of Neurobiology, Duke University, Durham, NC 27710 USA; 8 Department of Biomedical Engineering, Duke University, Durham, NC 27708 USA

**Keywords:** Parkinson's disease, Stem-cell research

## Abstract

Parkinson’s disease (PD) may optimally be treated with a disease-modifying therapy to slow progression. We compare data underlying surgical approaches proposed to impart disease modification in PD: (1) cell transplantation therapy with stem cell-derived dopaminergic neurons to replace damaged cells; (2) clinical trials of growth factors to promote survival of existing dopaminergic neurons; (3) subthalamic nucleus deep brain stimulation early in the course of PD; and (4) abdominal vagotomy to lower risk of potential disease spread from gut to brain. Though targeted to engage potential mechanisms of PD these surgical approaches remain experimental, indicating the difficulty in translating therapeutic concepts into clinical practice. The choice of outcome measures to assess disease modification separate from the symptomatic benefit will be critical to evaluate the effect of the disease-modifying intervention on long-term disease burden, including imaging studies and clinical rating scales, i.e., Unified Parkinson Disease Rating Scale. Therapeutic interventions will require long follow-up times (i.e., 5–10 years) to analyze disease modification compared to symptomatic treatments. The promise of invasive, surgical treatments to achieve disease modification through mechanistic approaches has been constrained by the reality of translating these concepts into effective clinical trials.

## What is disease modification in Parkinson’s disease?

Parkinson’s disease (PD) was originally described in 1817 by British physician James Parkinson in “Essay on the Shaking Palsy”^1^. PD pathology includes a loss of dopamine neurons in the substantia nigra pars compacta (SNc)^2–4^. Braak postulated that an unknown pathogen in the gut or nasal cavity could initiate the dopamine cell loss in sporadic PD through either the vagus nerve or olfactory tract^2^. Consistent with this postulate, a molecular alteration noted in sporadic PD, misfolded and aggregated α-synuclein (αS), may exhibit prion-like capability with the capability to cross synapses and spread within both the enteric and central nervous systems^3,5^. The complexity of PD origins, however, has been underscored by recent discussions of brain versus gut first etiology, molecular biomarkers, involvement of αS, phenotypic characteristics (such as unilateral vs bilateral, or tremor predominant vs akinetic-rigid vs postural instability, and gait disturbance), and genotyping^3,4,6,7^. These various biomarkers and characteristics may eventually be useful for surgery triage.

**Fig. 1:**
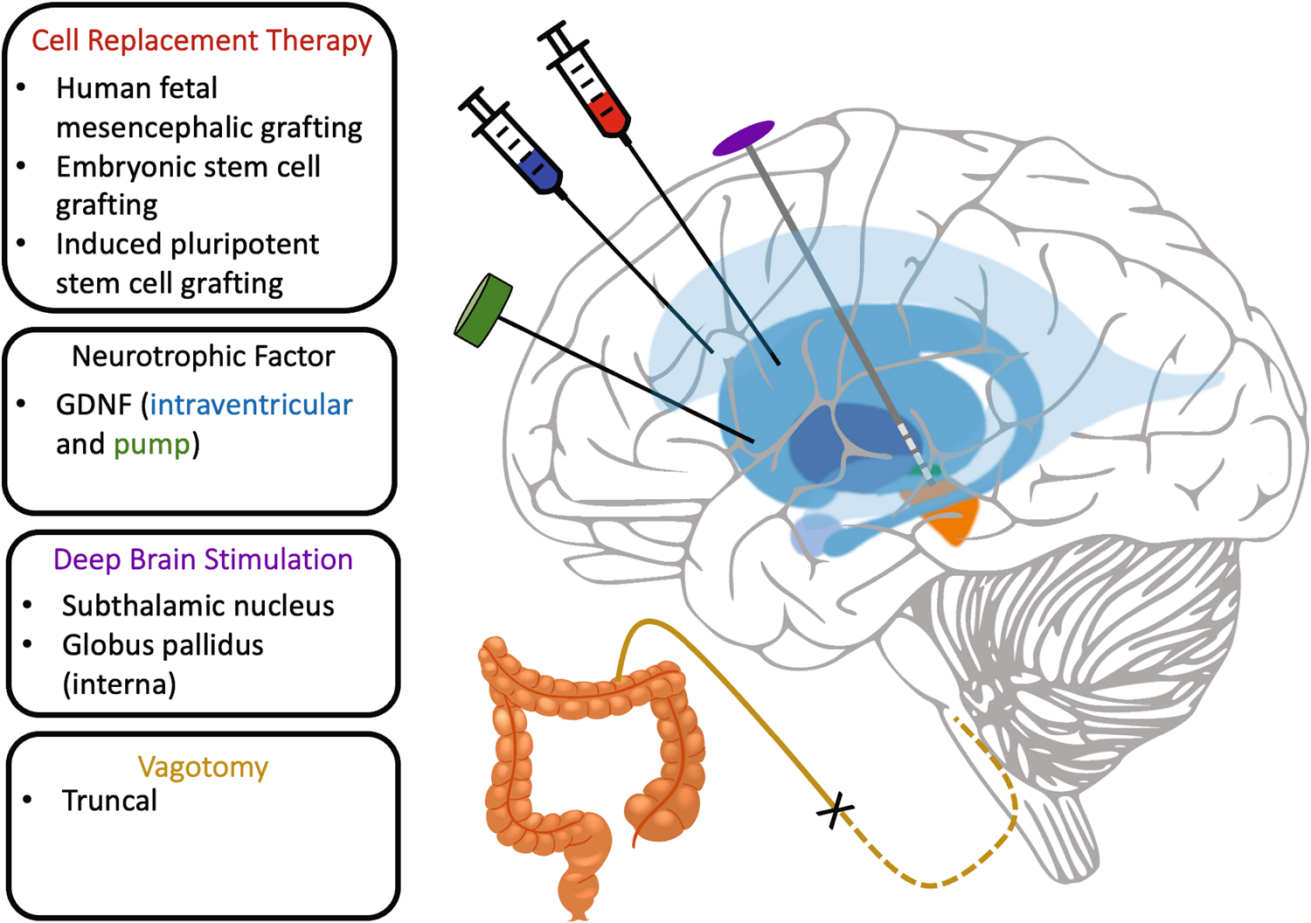
Surgical methods of disease modification. Disease-modifying surgical strategies include cell replacement therapy, infusion or gene therapy of dopamine neurotrophic growth factors (both intraventricular and intraparenchymal administration), early subthalamic deep brain stimulation, and abdominal vagotomy.

The concept of disease modification implies prevention or slowing of progression over a long-time scale (i.e., 5–10 years), potentially separate from a direct symptomatic treatment effect^8,9^. Surgical treatments promoted to confer possible disease modification include (1) cell transplantation (to replace lost dopaminergic neurons); (2) dopamine cell growth factor administration (to promote survival of and restore function in residual dopamine cells); (3) early, subthalamic deep brain stimulation (DBS) (to alleviate medication side effects, any associated toxicity, and induce growth factor release promoting neuronal survival); and (4) abdominal vagotomy (to prevent spread into the CNS of misfolded αS)^10^ (Fig. 1).

Our goal is to compare clinical outcomes of these surgical treatments proposed to alter disease progression in PD, to help prioritize future research (Table 1). There are multiple excellent reviews on medical approaches to disease modification in PD and appropriate clinical trial formats, but these have generally excluded surgical treatments^9,11^, whereas individual reviews of gene therapy, regenerative medicine strategies, and stem cell transplantation in PD^12,13^ do not critically discuss comparative potential efficacy and disease modification across surgical approaches^10^. Inherent to a discussion of surgical approaches is the trade-off between the greater invasiveness and risks associated with surgery compared to medical treatments, and whether this risk is truly balanced by a greater benefit than can be achieved by less invasive routes.

**Table 1:** Timing and outcome of surgical approaches.

PD treatment	PD timing	UPDRS III (%)	Hazard ratio
**Abdominal vagotomy**	>10 years prior	None	0.78^75^/0.58*^76^
**GDNF/Neurturin**	>5 years after	NS^39,40^	---------------
**Early Neurturin subset**	<5 years after	−14% *^40^	---------------
**Embryonic Transplants**	~14 years after	−18% *^19^/ NS^20^	---------------
**iPSC cells**	To be defined	---------	--------------
**STN/GPi DBS**	~10–13 years after	−32% *^65–67^	---------------
**Early STN DBS**	after PD diagnosis	NS^54^	0.31*^54^

Specific treatments are described in the left column, and the timing is with respect to the clinical diagnosis of Parkinson’s disease (PD). The optimal timing for abdominal vagotomy as a preventative measure would likely be >10 years prior to Parkinson’s diagnosis, so a cohort at risk defined by surrogate biomarkers may be essential to identify. Other treatments have generally been applied in the window of >4 years after diagnosis of Parkinson’s, similar to the current FDA approval for STN/GPi DBS. The pilot Early STN DBS trial is one of the few to gain FDA approval to proceed at the time of Parkinson’s diagnosis. The Hazard Ratio indicates protection against progression. NS indicates no significant difference vs placebo.

*Indicates statistical significance (at least *P* < 0.05 difference)

## Cell transplantation

Since degeneration of dopamine cells in the SNc underlies the pathogenesis of PD regardless of phenotype or genotype^2,6,7,14^ a natural consideration is simply to replace cells through grafting of dopamine neurons. The grafted cells need to integrate within the host and restore damaged neural circuits. Questions include which specific cell sources are suitable as appropriate donor cells, where to place the cells in the brain (i.e., directly into the SNc or into the target field in the putamen), and the mechanisms of action of the grafted cells^15^. Early clinical attempts employed autologous adrenal autografts^16^ but with only brief and modest improvement in motor symptoms, since the transplanted chromaffin cells do not differentiate into dopamine neurons. Alternatively, nonneural cells have not proven capable of either restoring dopaminergic circuits in PD or demonstrating efficacy^17^.

The grafting of embryonic dopaminergic neurons has demonstrated variable outcomes in human trials. The Lund group observed improvement in motor function and PET imaging following grafting of embryonic mesencephalic tissue into the striatum of PD patients^18^. Subsequently two double-blind clinical trials of transplantation therapy for PD (using mixed allograft embryonic tissue)^19,20^ demonstrated a net benefit, but some patients (Table 1) showed unexpected dyskinesia. Complicating issues in trial outcomes included the inherent neurodegenerative nature of PD, potential host-to-graft transfer of Lewy body pathology, difficulty with availability, quality control concerns, and embryonic allograft inconsistency^18,21,22^. A few long-term surviving PD patients were noted to have grafts on autopsy and to be less dependent on l-DOPA posttransplant^22,23^, suggesting a relationship between surviving grafted DA neurons and clinical benefits.

The advent of human pluripotent stem cells, including embryonic stem cells (ESCs) from blastocysts and induced pluripotent stem cells (iPSCs) reprogrammed from somatic cells, has rekindled the drive for cell replacement therapy^18,24,25^. Generation of authentic midbrain dopamine neurons from human ESCs and iPSCs has been shown to be feasible^26,27^. These generated dopamine neurons also are capable of surviving, projecting axons to target neurons, and correcting motor function deficits in rodent PD models^24,26,27^. The therapeutic effect of human iPSCs was further demonstrated in a nonhuman primate model of PD^28^. Hence, stem cell-based transplantation therapy shows promise and could overcome many of the limitations noted with embryonic dopamine cells^25,29–32^. Further, human iPSCs could undergo genome editing (i.e., clustered regularly interspaced short palindromic repeats [CRISPR] and CRISPR-associated protein 9 [Cas9]) for both disease model generation as well as to enhance the therapeutic potential once grafted^33,34^.

Though pilot human iPSC transplantation procedures have occurred^30,32^ there are numerous residual trial design, manufacturing, administration, and ethical questions that will need to be addressed before larger-scale controlled trials can commence^10,13,29^.

### Which PD patient population is ideal to consider?

Younger PD patients without tremor (i.e., <60 years) may be more responsive to cell transplantation since tremor did not reliably improve in previous cell transplantation studies^19,35^. Whether older patients with more severe symptoms, tremors, longer duration of disease, or less robust levodopa-responsiveness are also potential candidates for cell therapy is unclear^10^.

### Will the grafted dopamine neurons undergo the same pathological changes as the PD host?

PD pathology, such as mutant αS, may spread from diseased host cells to healthy grafted cells^21^. Autopsy analysis revealed Lewy bodies in a minority of grafted dopamine neurons^21^, ranging from 2% at 11 years, 5% at 16 years, to 11% at 24 years post-transplantation^23^. Therefore, this slow and low rate of Lewy body development in the grafted cells does not appear to substantially affect the potential benefit for decades to come.

### Which locations should be considered for grafting and how many cells?

Embryonic cell grafts have traditionally been injected into the striatum (i.e., caudate and putamen)^19,20^. Grafts in the striatum run the risks of less tight regulation of neurotransmitter release. Cells transplanted into the nigra are likely regulated appropriately but require long delays to project axons to the target striatum, as shown in a rodent model^24^. One may consider grafts to both the striatum and substantia nigra.

### Autologous vs. allogeneic transplant?

Human iPSC-derived dopamine cell transplant therapy has been demonstrated in a nonhuman primate model of PD^28^. Experience from embryonic transplants showed a variable graft survival without immunosuppression^19,20^. Hence, the current consensus is to treat the recipients with immune suppressants for a period (~1 year) for an allogeneic transplant. The use of a patient’s own iPSCs enables autologous transplant, which would avoid immune suppression but the process is time-consuming and costly. The latter may be overcome by the use of universal donor iPSCs, produced by knocking out components of the human leukocyte antigen^36^. On the other hand, allogeneic grafts are often walled off by the host tissue with less integration, as noted in embryonic grafts in PD patients and stem cell grafts in nonhuman primates^19,20,37^. In contrast, autologous grafts usually merge into the host tissue without a clear boundary and with extensive axonal outgrowth^37^.

### Efficacy and relative benefit

Typical disease outcome measures like Unified Parkinson Disease Rating Scale (UPDRS) are useful (as in most PD trials) but placebo effects and expectation of benefit may interfere with assessment^38–41^. An intermediate biomarker may help to gauge effective dopamine re-innervation of the striatum, such as Fluorodopa uptake or vesicular monoamine type 2 transporters (VMAT2) positron emission tomography (PET) scans^30,42^. There are cost concerns, based on decades of autologous and allogeneic cell therapy in patients undergoing bone marrow transplantation^43^. Relative treatment benefits may be measured in terms of quality-adjusted life-years (QALYs) and the incremental cost-effectiveness ratio^44^. Further feasibility and Phase II/III pivotal trials will clarify both relative efficacy and cost^10^. In these trials, outcome tools may be optimally assessed after withholding dopaminergic medications for a true baseline condition, including patient-oriented, objective, and biomarker measures^45^. It is worth noting investigators who are developing PD cell therapy, including the GForce-PD collaboration, regularly discuss steps and procedures critical for clinical application^13,35^.

### Summary of cell transplantation

Though promising and moderate efficacy was demonstrated in prior embryonic trials^19^ there are multiple issues to be resolved in pilot trial designs to achieve a reliable clinical outcome.

## Dopamine neurotrophic factors

Neural growth factors facilitate neuronal growth, survival, and maturation (i.e., dendritic and axonal elongation). The discovery of glial-derived neurotrophic factor (GDNF) in 1993 by Lin et al. demonstrated a survival factor specific to dopaminergic neurons^46^. Preclinical in vivo studies demonstrated the ability of GDNF to rescue and regenerate dopaminergic neurons, which led to open-label and randomized clinical trials^38,40,47–49^. However, initial intraventricular administration of GDNF^47^ demonstrated unanticipated side effects of nausea and paresthesias due to off-target effects on non-dopaminergic cell populations.

Hence, subsequent trials administered GDNF through either parenchymal infusions into the striatum^48^ or via gene therapy to transfect the host, but with overall negative results^10,40,50^. More recent trials of parenchymal GDNF infusions have shown improved delivery techniques to the striatum but echoed earlier trial results in which treatment effects were clouded by placebo responses^39^, a typical problem with randomized PD trials^41^. However, re-analysis of PD patients treated with a GDNF analog (Fig. 3 in Olanow et al.^40^), neurturin gene therapy, who were less than 5 years beyond PD diagnosis, did show a significant improvement compared to placebo (-14 points UPDRS-3, 40% change, Table 1)^40^. This result suggests that future trials be directed towards patients recently diagnosed with PD to gain the most benefit, since there may be more residual, functional dopamine cells in the SNc that could respond to the growth factor.

The lack of a consistent treatment benefit with growth factors regardless of administration approach has prompted a further review of regenerative therapy^10,38^. Growth factors are dependent on the presence of residual, functional dopamine cells, which rapidly decline in number as PD progresses, though if still alive could potentially be “rescued”^51^. Also, capability for retrograde axonal transport from the target field (i.e., putamen) back to the SNc is critical for growth factor function but this may be defective in humans with PD compared to a nonhuman primate model of PD^52^. Subsequent trials combined both putaminal and SNc injections of neurturin viral transfection to attempt to overcome this limitation^40^. These overall negative studies highlight the difficult process of translation from nonhuman primates into human clinical trials^12^. Additionally, most growth factor trials in Parkinson’s disease have shown a significant placebo effect over 1–2 years, implying that expectation of benefit may overwhelm any observed treatment response^39–41^, whereas other surgical PD trials (e.g., Vitek et al. pallidotomy trial) typically demonstrate a long-term progression of PD symptoms in control patients^53,54^. In addition to GDNF and neurturin, other analogous growth factors in the GDNF family may also eventually demonstrate benefit, particularly artemin and persephin, though these have not yet been tested in clinical trials^55,56^.

## Deep brain stimulation

Deep brain stimulation (DBS) for PD tremor was introduced by Benabid and colleagues in 1987^57^. Pallidotomy studies (Leksell posteroventral pallidotomy procedures)^58^ revealed that lesions in the internal segment of the globus pallidus (GPi) could help control symptoms of bradykinesia, rigidity, tremor, and dyskinesias^53,54^, while lesions in the subthalamic nucleus (STN) of the nonhuman primate 1-methyl-4-phenyl-1,2,3,6-tetrahydropyridine (MPTP) model of PD also markedly improved Parkinsonian symptoms^59^. This work rapidly led to the clinical adoption of DBS for PD^59^. The initial concept of how DBS worked centered on a “temporary lesion effect” induced during stimulation^57,60^. Subsequent studies, however, led to the notion that DBS mechanisms were more complicated, also activating outputs from the stimulated structure, leading to widespread modulation of basal ganglia-thalamocortical circuits and improving the downstream effects of dopaminergic deficiency in a non-chemical manner^60–64^.

Multiple randomized DBS studies of PD have confirmed symptomatic benefit^65–67^, now recommended to within four years of PD disease onset when medically indicated (medication refractory tremor, motor fluctuations, or medication limiting side effects)^68^. Treatment effects of DBS in PD are considered symptomatic, with expected PD disease progression occurring particularly in non-motor symptoms, such as worsening balance and gait, swallowing, speech, and cognition^65,67,69^. Overall, STN or GPi DBS therapy shows ~32% motor improvement in UPDRS III in patients ~10 years from PD diagnosis^44^.

Symptom improvement through DBS allows for the reduction of dopaminergic medication. Although levodopa toxicity to dopamine cells has been reported in vitro^70^ this finding has not been replicated in vivo hence the benefits of levodopa are felt to outweigh any theoretical toxicity^71^. Benefits of reductions of levodopa following DBS may be related more to the reduction in drug-induced dyskinesia, motor fluctuations, or mood/cognitive side effects associated with antiparkinsonian medication. The EARLY-STIM study compared active STN DBS at the time of symptomatic PD onset to optimal levodopa therapy [ODT]^54^. At the 5-year outcome assessment, off DBS and medical treatment for 7 days (avoiding long duration levodopa responses^71^), the patients who had undergone active STN DBS stimulation showed improved tremor compared to ODT, with a hazard ratio [HR] of 0.31 confirming tremor reduction (*p* < 0.001 vs. ODT). This finding suggests a disease-modifying effect on at least one symptom (tremor) in comparison to medical treatment^72^. However the numbers were small and a pivotal, multicenter trial will be needed to fully evaluate if early STN DBS can slow the progression of motor signs and symptoms of PD. Furthermore, the constant stimulation of STN excitatory neurons (at 130–180 Hz) may result in brain-derived neurotrophic factor (BDNF) release to STN and distant circuit targets, potentially exerting a significant neuroprotective effect^60,73^. Though STN may be an initial target for possible disease modification, GPi should not be excluded, given its projections to the brainstem and thalamocortical circuits that are involved in both motor and non-motor function.^74^

## Abdominal vagotomy

Abdominal vagotomy can improve refractory peptic ulcer disease^75,76^. Given the Braak hypothesis and the potential for mutant αS to be transferred from the gut to the CNS through the vagus nerve, early vagotomy prior to PD diagnosis, that severs the connection between the gut (particularly the colon) and the brainstem, could be considered as a disease-modifying or preventative approach to alter PD development. Two large population series compared truncal vagotomy (which severs the vagus nerve from the entire gut including the colon) and selective vagotomy (severing only stomach vagus nerve branches for peptic ulcer treatment)^75,76^. In a Swedish matched-cohort study, vagotomy overall was not associated with reduced PD risk (HR 0.96), but within the truncal vagotomy cohort, there was a suggested decreased risk of PD development (HR 0.78)^75^. In a Danish registry study, when compared to the general population (HR 0.53), and selective vagotomy (HR 0.58), the eventual risk of PD was significantly decreased in the truncal vagotomy cohort (see Table 1)^76^.

Similarly, preclinical models^5^ show that mutant αS originating in the gut can be transferred trans-synaptically through the vagus nerve to the central nervous system^3^. Additionally, gut wall injection of preformed, abnormal fibrils of αS led to the conversion of endogenous αS into the pathological phenotype^5^, resulting in the transfer of the pathologic αS into the central nervous system. A prospective evaluation of this intervention could include a randomized, clinical trial where younger individuals with risk factors suggestive of PD development later in life, such as mutant αS (identified via colonic biopsy^8^) or clinical predictors^77^, would undergo either truncal vagotomy or a placebo intervention at least 10 years prior to any PD diagnosis^8^. Vagotomy may lead to vomiting, diarrhea, and dysphagia^78^, which underscores the need for developing high probability predictive biomarkers of PD before consideration of such an invasive study.

## Comparison of approaches

These potential disease-modifying approaches to treat PD progression await definitive individual and comparative pivotal trials, preferably with trial formats optimized for detecting disease modification^9^. Outcome measures included in trials to establish disease modification should include disease state assessment over 5–10 years while temporarily off all confounding symptomatic treatments (i.e., DBS and PD medications) with both appropriate clinical scales (i.e., UPDRS) as well as more innovative biomarkers^8,10,45^. Biomarkers could include Fluorodopa uptake, VMAT2 PET, and other markers of dopamine innervation in the striatum^3,8^. Disease modification may target progression of PD through multiple mechanisms^18,25,29,31,35,38,49^. For example, other gene therapy trials have targeted restoration of dopaminergic function by delivering one or more gene(s) necessary for dopamine conversion from levodopa, such as l-amino acid decarboxylase [AADC], into nondegenerating striatal neurons, the ultimate site of dopamine action^79^. In this open-label, phase I study, reduced requirements for PD medications (21–30%) were noted but further, randomized trials will be needed to confirm the potential, disease modification benefit.

Common metrics for determining relative efficacy amongst different medical and surgical treatment approaches should be prospectively identified so that similar outcomes can be assessed. Once initial trial information is available, the possibility of direct, comparative efficacy trials across treatments and modalities should also be considered. It remains to be determined if the degree of disease modification afforded by these invasive surgical procedures can effectively offset the associated risks of performing these treatments in PD patients. However, if potential treatments are not explored with critical trials and due diligence, we will continue to ask the same questions 10, 20, or 30 years from now, with persistent claims of “promise” rather than demonstrated efficacy. These approaches highlight the difficulty in translating preclinical mechanistic concepts into effective, practical clinical treatments.

## Methods

We review surgical outcomes from existing literature and no individual patients are involved, hence there are no relevant patient consent issues, Institutional Review Board involvement, or ethics approval integral to this perspective. The initial search terms used were: surgical, Parkinson’s disease, disease modification, cell transplantation, dopamine growth factors, deep brain stimulation, abdominal vagotomy. However, most articles found with these search terms did not discuss surgical approaches to disease modification, necessitating reviewing each article for context. Additionally, we emphasize clinical trial outcomes involving surgical approaches within the last 20 years since this is a focused perspective.

### Reporting Summary

Further information on research design is available in the Nature Research Reporting Summary linked to this article.

## Supplementary information

Reporting Summary Checklist

## Data Availability

All data discussed in this article arise from prior published articles which are freely available in public library databases such as PubMed.

## References

[1] Parkinson J (2002). An essay on the shaking palsy. 1817. J. Neuropsychiatry Clin. Neurosci..

[2] Braak H, Del Tredici K (2017). Neuropathological staging of brain pathology in sporadic Parkinson’s disease: separating the wheat from the chaff. J. Parkinsons Dis..

[3] Breen DP, Halliday GM, Lang AE (2019). Gut-brain axis and the spread of α-synuclein pathology: vagal highway or dead end?. Mov. Disord..

[4] Fearon C, Lang AE, Espay AJ (2021). The logic and pitfalls of Parkinson’s disease as “brain-first” versus “body-first” subtypes. Mov. Disord..

[5] Kim S (2019). Transneuronal propagation of pathologic α-synuclein from the gut to the brain models Parkinson’s disease. Neuron.

[6] Blesa, J., Foffani, G., Dehay, B., Bezard, E. & Obeso, J. A. Motor and non-motor circuit disturbances in early Parkinson disease: which happens first? *Nat. Rev. Neurosci*. 10.1038/s41583-021-00542-9 (2021). 10.1038/s41583-021-00542-9 34907352

[7] Borghammer P (2021). The α-synuclein origin and connectome model (SOC Model) of Parkinson’s disease: explaining motor asymmetry, non-motor phenotypes, and cognitive decline. J. Parkinsons Dis..

[8] Espay AJ (2020). Disease modification and biomarker development in Parkinson disease: revision or reconstruction?. Neurology.

[9] Vijiaratnam N, Simuni T, Bandmann O, Morris HR, Foltynie T (2021). Progress towards therapies for disease modification in Parkinson’s disease. Lancet Neurol..

[10] Buttery, P. C. & Barker, R. A. Gene and cell-based therapies for Parkinson’s disease: where are we? *Neurotherapeutics*10.1007/s13311-020-00940-4 (2020). 10.1007/s13311-020-00940-4 PMC7598241 33128174

[11] Poewe W, Seppi K, Marini K, Mahlknecht P (2020). New hopes for disease modification in Parkinson’s disease. Neuropharmacology.

[12] Hitti FL, Yang AI, Gonzalez-Alegre P, Baltuch GH (2019). Human gene therapy approaches for the treatment of Parkinson’s disease: an overview of current and completed clinical trials. Parkinsonism Relat. Disord..

[13] Barbuti, P. A. et al. Recent advances in the development of stem-cell-derived dopaminergic neuronal transplant therapies for Parkinson’s disease. *Mov. Disord.*10.1002/mds.28628 (2021). 10.1002/mds.28628 33963552

[14] Braak H (2003). Staging of brain pathology related to sporadic Parkinson’s disease. Neurobiol. Aging.

[15] Parmar M, Torper O, Drouin-Ouellet J (2019). Cell-based therapy for Parkinson’s disease: a journey through decades toward the light side of the Force. Eur. J. Neurosci..

[16] Goetz CG (1989). Multicenter study of autologous adrenal medullary transplantation to the corpus striatum in patients with advanced Parkinson’s disease. N. Engl. J. Med..

[17] Conese M (2019). Harnessing stem cells and neurotrophic factors with novel technologies in the treatment of Parkinson’s disease. Curr. Stem Cell Res. Ther..

[18] Lindvall O (2016). Clinical translation of stem cell transplantation in Parkinson’s disease. J. Intern. Med..

[19] Freed CR (2001). Transplantation of embryonic dopamine neurons for severe Parkinson’s disease. N. Engl. J. Med..

[20] Olanow CW (2003). A double-blind controlled trial of bilateral fetal nigral transplantation in Parkinson’s disease. Ann. Neurol..

[21] Kordower JH, Chu Y, Hauser RA, Freeman TB, Olanow CW (2008). Lewy body-like pathology in long-term embryonic nigral transplants in Parkinson’s disease. Nat. Med..

[22] Kordower JH (2017). Robust graft survival and normalized dopaminergic innervation do not obligate recovery in a Parkinson disease patient. Ann. Neurol..

[23] Li W (2016). Extensive graft-derived dopaminergic innervation is maintained 24 years after transplantation in the degenerating parkinsonian brain. Proc. Natl Acad. Sci. USA.

[24] Xiong, M. et al. Human stem cell-derived neurons repair circuits and restore neural function. *Cell Stem Cell*10.1016/j.stem.2020.08.014 (2020). 10.1016/j.stem.2020.08.014 PMC7796915 32966778

[25] Zhang Q, Chen W, Tan S, Lin T (2017). Stem cells for modeling and therapy of Parkinson’s dDisease. Hum. Gene Ther..

[26] Kriks S (2011). Dopamine neurons derived from human ES cells efficiently engraft in animal models of Parkinson’s disease. Nature.

[27] Kirkeby A (2012). Generation of regionally specified neural progenitors and functional neurons from human embryonic stem cells under defined conditions. Cell Rep..

[28] Kikuchi T (2017). Human iPS cell-derived dopaminergic neurons function in a primate Parkinson’s disease model. Nature.

[29] Jankovic J, Okun MS, Kordower JH (2020). Stem cells: scientific and ethical quandaries of a personalized approach to Parkinson’s disease. Mov. Disord..

[30] Schweitzer JS (2020). Personalized iPSC-derived dopamine progenitor cells for Parkinson’s disease. N. Engl. J. Med..

[31] Sonntag KC (2018). Pluripotent stem cell-based therapy for Parkinson’s disease: current status and future prospects. Prog. Neurobiol..

[32] Takahashi J (2019). Preparing for first human trial of induced pluripotent stem cell-derived cells for Parkinson’s disease: an interview with Jun Takahashi. Regen. Med..

[33] Guan, L. et al. CRISPR-Cas9-mediated gene therapy in neurological disorders. *Mol. Neurobiol*. 10.1007/s12035-021-02638-w (2021). 10.1007/s12035-021-02638-w 34813019

[34] McTague A, Rossignoli G, Ferrini A, Barral S, Kurian MA (2021). Genome editing in iPSC-based neural systems: from disease models to future therapeutic strategies. Front. Genome Ed..

[35] Barker RA (2019). Designing stem-cell-based dopamine cell replacement trials for Parkinson’s disease. Nat. Med..

[36] Xu H (2019). Targeted disruption of HLA genes via CRISPR-Cas9 generates iPSCs with enhanced immune compatibility. Cell Stem Cell.

[37] Tao Y (2021). Autologous transplant therapy alleviates motor and depressive behaviors in parkinsonian monkeys. Nat. Med..

[38] Barker RA (2020). GDNF and Parkinson’s disease: where next? A summary from a recent workshop. J. Parkinsons Dis..

[39] Whone AL (2019). Extended treatment with glial cell line-derived neurotrophic factor in Parkinson’s disease. J. Parkinsons Dis..

[40] Warren Olanow C (2015). Gene delivery of neurturin to putamen and substantia nigra in Parkinson disease: a double-blind, randomized, controlled trial. Ann. Neurol..

[41] Mestre, T. A. et al. Expectations of benefit in a trial of a candidate disease-modifying treatment for Parkinson disease. *Mov. Disord.*10.1002/mds.28630 (2021). 10.1002/mds.28630 33942376

[42] Siderowf A (2014). PET imaging of amyloid with Florbetapir F 18 and PET imaging of dopamine degeneration with 18F-AV-133 (florbenazine) in patients with Alzheimer’s disease and Lewy body disorders. BMC Neurol..

[43] Rowe JM (1994). Recommended guidelines for the management of autologous and allogeneic bone marrow transplantation. A report from the Eastern Cooperative Oncology Group (ECOG). Ann. Intern. Med..

[44] Mahajan, U. V. et al. Bilateral deep brain stimulation is the procedure to beat for advanced Parkinson disease: a meta-analytic, cost-effective threshold analysis for focused ultrasound. *Neurosurgery*10.1093/neuros/nyaa485 (2020). 10.1093/neuros/nyaa485 PMC8190460 33295629

[45] Polgar S, Karimi L, Buultjens M, Morris ME, Busse M (2018). Assessing the efficacy of cell transplantation for Parkinson’s disease: a patient-centered approach. J. Parkinsons Dis..

[46] Lin LF, Doherty DH, Lile JD, Bektesh S, Collins F (1993). GDNF: a glial cell line-derived neurotrophic factor for midbrain dopaminergic neurons. Sci.ence.

[47] Nutt JG (2003). Randomized, double-blind trial of glial cell line-derived neurotrophic factor (GDNF) in PD. Neurology.

[48] Lang AE (2006). Randomized controlled trial of intraputamenal glial cell line-derived neurotrophic factor infusion in Parkinson disease. Ann. Neurol..

[49] Gash DM, Gerhardt GA, Bradley LH, Wagner R, Slevin JT (2020). GDNF clinical trials for Parkinson’s disease: a critical human dimension. Cell Tissue Res..

[50] Marks WJ (2010). Gene delivery of AAV2-neurturin for Parkinson’s disease: a double-blind, randomised, controlled trial. Lancet Neurol..

[51] Kordower JH (2013). Disease duration and the integrity of the nigrostriatal system in Parkinson’s disease. Brain.

[52] Kordower JH (2000). Neurodegeneration prevented by lentiviral vector delivery of GDNF in primate models of Parkinson’s disease. Science.

[53] Vitek JL (2003). Randomized trial of pallidotomy versus medical therapy for Parkinson’s disease. Ann. Neurol..

[54] Hacker ML (2020). Deep brain stimulation in early-stage Parkinson disease: five-year outcomes. Neurology.

[55] Yin XF (2015). Lentivirus-mediated Persephin over-expression in Parkinson’s disease rats. Neural. Regen. Res..

[56] Zhu S (2020). The role of glial cell line-derived neurotrophic factor family member artemin in neurological disorders and cancers. Cell Prolif..

[57] Benabid AL, Chabardes S, Mitrofanis J, Pollak P (2009). Deep brain stimulation of the subthalamic nucleus for the treatment of Parkinson’s disease. Lancet Neurol..

[58] Laitinen LV, Bergenheim AT, Hariz MI (1992). Leksell’s posteroventral pallidotomy in the treatment of Parkinson’s disease. J. Neurosurg..

[59] Bergman H, Wichmann T, DeLong MR (1990). Reversal of experimental parkinsonism by lesions of the subthalamic nucleus. Science.

[60] Hashimoto T, Elder CM, Okun MS, Patrick SK, Vitek JL (2003). Stimulation of the subthalamic nucleus changes the firing pattern of pallidal neurons. J. Neurosci..

[61] Brocker, D. T. et al. Optimized temporal pattern of brain stimulation designed by computational evolution. *Sci. Transl. Med.*10.1126/scitranslmed.aah3532 (2017). 10.1126/scitranslmed.aah3532 PMC5516784 28053151

[62] Schmidt SL, Brocker DT, Swan BD, Turner DA, Grill WM (2020). Evoked potentials reveal neural circuits engaged by human deep brain stimulation. Brain Stimul..

[63] Johnson MD, Vitek JL, McIntyre CC (2009). Pallidal stimulation that improves parkinsonian motor symptoms also modulates neuronal firing patterns in primary motor cortex in the MPTP-treated monkey. Exp. Neurol..

[64] Wang J (2017). Network-wide oscillations in the parkinsonian state: alterations in neuronal activities occur in the premotor cortex in parkinsonian nonhuman primates. J. Neurophysiol..

[65] Deuschl, G. et al. Comparing two randomized deep brain stimulation trials for Parkinson’s disease. *J. Neurosurg.*10.3171/2018.12.Jns182042 (2019). 10.3171/2018.12.JNS182042 30952118

[66] Odekerken VJ (2016). GPi vs STN deep brain stimulation for Parkinson disease: three-year follow-up. Neurology.

[67] Vitek JL (2020). Subthalamic nucleus deep brain stimulation with a multiple independent constant current-controlled device in Parkinson’s disease (INTREPID): a multicentre, double-blind, randomised, sham-controlled study. Lancet Neurol..

[68] Schuepbach WM (2013). Neurostimulation for Parkinson’s disease with early motor complications. N. Engl. J. Med..

[69] Limousin P, Foltynie T (2019). Long-term outcomes of deep brain stimulation in Parkinson disease. Nat. Rev. Neurol..

[70] Giannopoulos S, Samardzic K, Raymond BBA, Djordjevic SP, Rodgers KJ (2019). L-DOPA causes mitochondrial dysfunction in vitro: a novel mechanism of L-DOPA toxicity uncovered. Int. J. Biochem. Cell Biol..

[71] Poewe W, Espay AJ (2020). Long duration response in Parkinson’s disease: levodopa revisited. Brain.

[72] de Bie RMA, Clarke CE, Espay AJ, Fox SH, Lang AE (2020). Initiation of pharmacological therapy in Parkinson’s disease: when, why, and how. Lancet Neurol..

[73] Fischer DL, Sortwell CE (2019). BDNF provides many routes toward STN DBS-mediated disease modification. Mov. Disord..

[74] Kim J (2017). Inhibitory basal ganglia inputs induce excitatory motor signals in the thalamus. Neuron.

[75] Liu B (2017). Vagotomy and Parkinson disease: a Swedish register-based matched-cohort study. Neurology.

[76] Svensson E (2015). Vagotomy and subsequent risk of Parkinson’s disease. Ann. Neurol..

[77] Searles Nielsen S (2017). A predictive model to identify Parkinson disease from administrative claims data. Neurology.

[78] Skellenger ME, Jordan PH (1983). Complications of vagotomy and pyloroplasty. Surg. Clin. North Am..

[79] Christine CW (2022). Safety of AADC gene therapy for moderately advanced Parkinson disease: three-year outcomes from the PD-1101 trial. Neurology.

